# Addressing Non-Communicable Diseases in Primary Healthcare in Kyrgyzstan: A Study on Population’ Knowledge and Behavioral Changes

**DOI:** 10.3389/ijph.2023.1605381

**Published:** 2023-07-04

**Authors:** Elvira Muratalieva, Mehrigiul Ablezova, Tolkun Djamangulova, Tobias Hoffarth, Katarzyna Kissimova-Skarbek, Silke Graeser, Mathieu Nendaz, David Beran

**Affiliations:** ^1^ Swiss Development and Cooperation in the Kyrgyz Republic, Bishkek, Kyrgyzstan; ^2^ Department of Sociology, American University of Central Asia, Bishkek, Kyrgyzstan; ^3^ Public Association “Healthy Future”, Bishkek, Kyrgyzstan; ^4^ GFA Consulting Group GmbH, Hamburg, Germany; ^5^ Institute of Public Health, Faculty of Health Sciences, Jagiellonian University Medical College, Krakow, Poland; ^6^ Euro Health Group, Soborg, Denmark; ^7^ Unit of Development and Research in Medical Education (UDREM), Department of General Internal Medicine, Faculty of Medicine of the University of Geneva, Geneva, Switzerland; ^8^ Division of Tropical and Humanitarian Medicine, University Hospitals and University of Geneva, Geneva, Switzerland

**Keywords:** NCDs, primary healthcare, PEN-protocol, health education, behaviour change

## Abstract

**Objective:** Non-communicable diseases (NCDs) in Kyrgyzstan are responsible for 83% of all deaths. This study aimed to assess the effectiveness of WHO “Package of Essential Interventions on NCDs” (PEN) on health education and counselling at primary healthcare in Kyrgyzstan.

**Methods:** Interventions consisted of information diffusion in primary care facilities and in communities by trained volunteers. The study aimed to assess the evolution of population’s knowledge and behaviour through a questionnaire applying a quasi-experimental approach. The sample size was 2,000 at baseline and after 4 years in intervention and control oblasts.

**Results:** Population’s knowledge and behaviour improved in intervention areas compared to control areas. Knowledge on NCD-related risks increased from 61% to 87%. Behaviour improved with physical activity increasing from 23% to 32%; smokers reduced from 22% to 20%; alcohol consumption reduced from 23% to 16%; daily walking (minimum 30’) improved from 40% to 71%.

**Conclusion:** This study suggests that the PEN-protocol is effective in improving healthy behaviour, thus potentially contributing to prevent NCDs. This example from Kyrgyzstan provides a practical example for promoting PEN-protocol adaptation in other countries.

## Introduction

Globally, non-communicable diseases (NCDs) kill 41 million people each year, equivalent to 71% of all deaths [1]. Environmental, occupational and behavioral risk factors are the main contributing factors to the increasing prevalence of NCD’s globally [1]. Major risk factors for NCDs are attributed to the individual behavioral issues such as tobacco use, physical inactivity, the harmful use of alcohol and unhealthy diets [1]. The Global Burden of Disease Study found that the magnitude of behavioral risks are second highest after the environmental risks [2] and concluded that population had not changed behaviors over 10 year period in particular related to diet, caloric intake, and physical activities.

NCDs in Kyrgyzstan are responsible for 83% of all deaths [3, 4]. Similar to global trends, the major risk factors for NCDs in Kyrgyzstan are mainly attributed to individual behavioral risks. According to the STEPwise approach to surveillance (STEPS), 25.7% of the Kyrgyz population smoke, 31.4% use alcohol, 83.7% of the population are physically inactive, 74% of population are not consuming the recommended quantity of fruits and vegetables, 21% are adding salt in their food before eating [5]. Main risk factors leading to the most of the death in Kyrgyzstan are the behavior related risks, where unhealthy diet and tobacco use are prevailing [6]. Nutrition habits of the Kyrgyz population are predominantly unhealthy [7] with prevailing carbohydrates, salty food and animal fat. All these worsened by limited physical activity of the population and resulting in high body mass-index. These risk factors are main contributors to the increasing prevalence of NCDs in Kyrgyzstan. Until 2018, the Kyrgyz Government did not consider any targeted intervention to address these risk factors.

All this evidence should inform decisions to introduce appropriate strategies to protect the health of the population. WHO defined that the most important way of reducing deaths from NCDs is to control unhealthy lifestyles, which includes reducing the use of tobacco and the harmful use of alcohol, maintaining an active lifestyle, and developing a healthy diet. Against this background, in 2010 WHO published a global strategy to strengthen Primary Healthcare (PHC) to better address NCDs using a protocol called “Package of Essential Non-communicable (PEN) Disease Interventions for Primary Healthcare in Low-Resource Settings” [3]. The PEN protocol is an innovative and action-oriented set of cost-effective interventions that can be delivered in low-resources settings with a focus on early detection of NCDs and promotion of healthy behavior among the population. The PEN protocol is designed to strengthen national capacities to integrate and scale up healthcare for heart diseases, stroke, diabetes, cancer, asthma, and chronic obstructive pulmonary diseases in PHC systems in low-resource countries. One of the PEN is specifically designed to tackle behavioral risk factors through counselling for healthy lifestyles, which includes: being physically active, eating a “heart healthy” diet and stopping tobacco, and avoiding harmful use of alcohol. In Kyrgyzstan, the PEN protocol was adapted in 2018 by the Ministry of Health and is implemented as a tool for the primary healthcare workers to better manage NCDs. To evaluate the results of the PEN implementation in Kyrgyzstan the Knowledge, Attitude and Practice (KAP) study was conducted during period of 2018–2021.

## Methods

### Context

**FIGURE 1:**
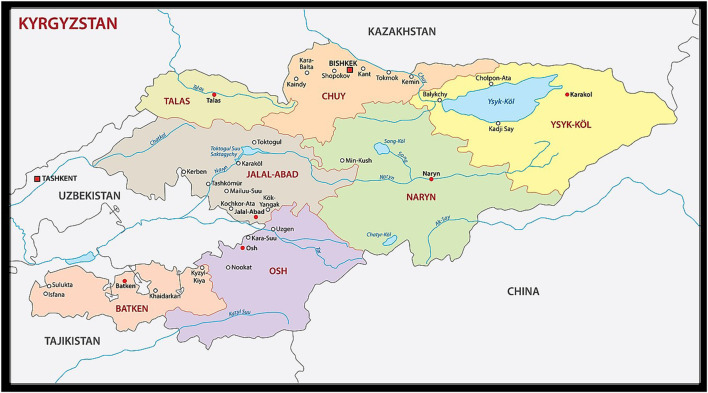
Map of Kyrgyzstan. (Addressing non-communicable diseases in primary health care in Kyrgyzstan: a study on population’ knowledge and behavioral changes; Naryn, Talas, Chui, Issyk-Kul and Djalalabad oblasts, Kyrgyzstan, 2018 and 2021).

Kyrgyzstan is located in Central Asia with a total population of 6.6 million as of 1st January 2021 (Figure 1, Map of Kyrgyzstan). In 2021 the population structure was the following one: 34.6% of the total population were children and adolescents, 57.1% were of working age, and 8.3% of retirement age. Life expectancy is 71.7 years (males 67.8 and females 76 years). The GDP *per capita* in 2021 was 1,023 USD and the Government is allocating around 2 USD annually per person for health services. This level of funding can cover only basic health services at primary care level. Other services are mostly covered by the population with almost 54% of out-of-pocket payment [6].

### Intervention

To address the common NCD risk factors in the Kyrgyz population, the PEN protocol was implemented in 2018 in four northern oblasts (Naryn, Talas, Chui and Issyk-Kul) with the aim to inform population on the following topics: practice regular physical activity, eat a “heart healthy” diet, stop tobacco, avoid harmful use of alcohol, and attend regular medical follow-up.

The adaptation of the PEN protocol was followed by the elaboration and delivery of a training module for primary healthcare workers (590 family doctors and 1,738 nurses) and 821 members of the Village Health Committees (network of volunteers). The PEN protocol implementation included a minimum 10-min discussion with patients regarding the specific NCD-related risk factors. Due to the lack of family doctors, it was decided to delegate this task to nurses at PHC facilities. This was done by creating a separate room where nurse could receive patients. Nurses received patients before their visit to a family doctor and collected all required measurements (height, weight) and information on nutrition and other habits, and calculated estimated NCD risks based on the provided formula in the PEN protocol. With this information, the patient then went to the family doctor for further consultation. Family doctors responsible for the diagnostic, management and treatment of NCDs with additional counselling on behavioural aspects. As part of NCD management, the Patients Education Schools were created in every PHC facility led by nurses. At this stage, these schools aimed at conducting group sessions for patients with hypertension and diabetes to better control their diseases. Every months these groups meet at the PHC facility to discuss methods to control their diseases and prevent complications. For the general population, the Public Health Centre produced promotional information on NCD-related risk factors that was distributed every quarter by the Village Health Committees and published on social media. In addition to this, specific health promotion tool with focus on NCD-related risk factors was elaborated for schoolchildren. To deliver this course, around 10 teachers per school were trained to conduct interactive trainings with schoolchildren. Overall, the intervention lasted for 4 years. The PEN implementation was fully integrated into the PHC system’s practice and did not require additional funding, except for the initial training of the personnel supported by the Swiss Development Cooperation. A Knowledge, Attitude, and Practice (KAP) study was carried out to assess the effect of the PEN protocol on the population’s knowledge, attitudes, and behaviour changes with regard to NCD-related risk factors such as nutrition, physical activity, tobacco, and alcohol consumption.

### Sampling and Data Collection

The KAP study started at the end of April 2018 with the collection of baseline data and ended in April 2021 with the collection of endline data. The methodology used for this KAP study in Kyrgyzstan was adapted from a similar approach developed in Mongolia in 2010 [8]. A quasi-experimental study design was used to compare the intervention areas and the control area. The intervention areas were Chui, Issyk-Kul, Naryn and Talas oblasts that are located in the northern part of Kyrgyzstan. The control area was Djalal-Abad oblast located in the southern part of the country, selected as a control because it is close to the intervention areas in terms of size, remoteness and health service level.

A questionnaire (Supplementary Annex S2) was used to collect NCD-related risk factors knowledge (15 questions) and self-reported practice about healthy diet, physical activity, tobacco and alcohol consumption (15 questions). The same questionnaire was used to collect data at the beginning and at the end of the intervention. It was developed in Russian and Kyrgyz languages, and took on average 15 min to be completed.

The target population of the study comprised all adults aged 18+ in five oblasts of Kyrgyzstan. The overall sample size in the baseline and endline studies was 2000 equally allocated across the oblasts (400 respondents per oblast). The samples were considered independent as in different years respondents were selected from different villages in the control and intervention areas. Regarding representativeness of the sample, for around 4 million population leaving in these 5 oblasts the selected group of 2,000 people is considered as representative. The sample composition followed proportional representation of the population by gender, age and residing area. In the data sampling a self-selection bias can be observed as individuals allowed to choose whether they want to participate in a research study.

KoBoToolBox (https://kf.kobotoolbox.org/#/forms/aefHE7PjAZrgYeQby7rpmm/data/table) software was used to conduct interviews during the data collection processes. The questionnaire was uploaded in this software, which was installed in tablets provided for data collection. In total, 16 staff members of the Health Promotion Units (HPU), a structure located within the Primary Healthcare Facilities at the rayon level, were involved to conduct the interviews in the intervention and control oblasts. To interview 400 people in each oblasts, HPU personnel proportionally divided the required number of people to the number of villages in their oblast. In average, 3 to 5 people per town and village were selected based on their availability and invited for interview. From gender perspective, the interviewers tried to ensure equal representation of women and men.

### Analysis

The outcomes of the study were aggregated in two main variables: 1) knowledge about NCD-related risk factors, 2) behaviour change in practice on physical activity, diet, tobacco and alcohol consumption. Empirical data were weighted to reflect population size of intervention area. Missing data were excluded from the results as they represented less than 1% for most questions.

Pearson’s chi-square test was used to compare baseline and endline proportions of participants aligned with the knowledge and behavioural indicators and analyse the association with intervention or control areas.

In case positive changes were observed both in intervention and control areas, the interaction effect in binary logistic regression was generated to estimate the strengths of difference in values (baseline vs. endline studies) in intervention and control areas.

All statistical analysis was performed with IBM SPSS (Statistical Package for the Social Sciences https://www.ibm.com/analytics/spss-statistics-software) Statistics 27. A *p*-value <0.05 was considered statistically significant (see Supplementary Annex S3).

## Results

The characteristics of participants are detailed in Supplementary Annex Table S1.

Demographically, in the study the share of urban population was slightly larger in the control area (19% in intervention area vs. 23% in control area). The proportion of males and females was similar in the control area whereas in the intervention area females (51%) slightly outnumbered the males (49%). The sampled population in the intervention areas was slightly younger than in the control area (26% or 416 people in intervention area of 18–28 years versus 28% or 112 people of the same age in control area, and 25% versus 31% of 29–38 years). The share of never-married respondents in the control area was smaller compared to the intervention area.

From education point of view, only a minority of respondents had basic and lower education, while those with secondary education were more numerous in the control area and those with higher education more numerous in the intervention area.

From employment view, more than half of the sample was not engaged in productive work and were either housewives, pensioners, unemployed, students or did not work due to disabilities. The share of farmers, private entrepreneurs and businessmen in the intervention area was higher than in the control one.

### Effect of the PEN on Knowledge About NCDs-Related Risk Factors

The endline study results showed that the overall level of knowledge on NCD-related risk factors improved in intervention and control areas (Supplementary Annex Table S2). The share of respondents who could not provide any answer to the asked questions in the questionnaire decreased in 2021 (7% in intervention and 4% in control area) compared with 2018 (15% and 35%, respectively).

Stress as an NCD risk factor was the leading answer in 2018 and remained the most frequent answer among respondents in the control area. According to data collected in intervention area, the knowledge of people had changed: they indicated more frequently factors that could be altered such as eating food with a high fat content (32% in 2018 and 68% in 2021), use of alcohol (29% in 2018 and 64% in 2021), smoking (28% in 2018 and 64% in 2021), eating salty (18% in 2018 and 61% in 2021) and high sugar food (18% in 2018 and 59% in 2021) as well as physical inactivity (11% in 2018 and 40% in 2021).

Chi-square test of independence was applied to assess the relationship between baseline and endline data from intervention and control area. People in the intervention area were more likely to mention at least one NCD risk factor in 2021 than in 2018 [X2 (1, N = 3,200) = 283.6, *p* < 0.0001]. The logistic regression illustrates that the difference in knowledge of NCD risk factors between the baseline and endline studies was significantly stronger in intervention than in control area (see Supplementary Annex Box S1). At the endline people in intervention area have a higher chance (by 82.4%) than people from control area to improve the knowledge about the NCDs risk factors (OR = 1.824, 95% CI [1.487, 2.161]). In the intervention areas, population’ knowledge about NCD risk factors is higher than in control area: it increased from 61% in 2018 to 87% in 2021.

### Effect of the PEN Protocol on Changing Behaviour in Practice

#### Physical Activity

The study’s results demonstrated (Supplementary Annex Table S3) that the level of self-reported physical activity in intervention area considerably improved. The share of those who reported doing sports increased from 23% in 2018 to 32% in 2021 and the share of those who walked no less 30 min per day increased from 40% in 2018 to 71% in 2021. It should be noted here that in control area, the proportion of those who walk daily decreased from 78% in 2018 to 61% in 2021. Reason behind this decrease could be influence of COVID-19, but this should be further investigated. The share of physically inactive respondents in intervention area halved in rural areas (58% in 2018 and 27% in 2021) and among males (56% in 2018 and 26% in 2021). The relation between these variables was significant, X^2^ (1, N = 3,200) = 316.8, *p* < 0.0001. People in intervention area were more physically active (were more likely to walk daily) in 2021 than in 2018.

#### Smoking

The share of smokers reduced from 22% in 2018 to 20% in 2021 (Supplementary Annex Table S4). Whereas the share of smokers in the control oblast increased from 12% in 2018 to 15% in 2021. It is worth noting, however, that smoking was still more prevalent in the intervention areas than in control region. Lower prevalence of smokers in the control area could be explained by influence of religion, which is much higher in the southern regions. However, the fact that prevalence of smoking population in the control area increased over 4 years should be further investigated. A chi-square test of independence was performed to examine the relation between the year of study (baseline or endline) and smoking behaviour of people in intervention area. The relation between these variables was not statistically significant, X^2^ (1, N = 3,199) = 1.1, *p* = 0.298. The reduction of the share of smokers in intervention area in 2021 compared to 2018 might be obtained by chance. It is interesting to note that the share of smokers increased in urban settlements both in intervention and control areas. However, the increase in numbers of smokers during the 4-year period 2018–2021 in intervention area is not statistically significant [X^2^ (1, N = 638) = 0.132, *p* = 0.716]. In addition, the share of smokers among females in intervention area increased from 2% in 2018 to 3% in 2021, however, this increase was not statistically significant as well [X^2^ (1, N = 3,199) = 1.48, *p* = 0.225].

#### Alcohol Consumption

The number of respondents who consumed alcohol in the control area within the past 30 days slightly increased from 10% in 2018 to 11% 2021. In particular, increase of alcohol consumption in the control area was considerable (from 4% in 2018 to 16% in 2021) among urban respondents. The share of those who consumed alcohol in intervention area, decreased from 23% in 2018 to 16% in 2021. A chi-square test of independence was performed to examine the relation between the year of study (baseline or endline) and alcohol consumption of people in intervention area. The relation between these variables was statistically significant, X^2^ (1, N = 3,200) = 20.4, *p* < 0.0001. The share of alcohol consumers in intervention area has significantly reduced in 2021 compared to 2018. Alcohol consumption has reduced among rural and urban inhabitants as well as among males and females in intervention area. Considerable increase in the number of those who consume alcohol in control area was observed in urban settlements and among males. Reasons behind this increase require another study to explain this result.

### Diet

#### Fruits and Vegetable Consumption

The longitudinal data obtained during the baseline and endline demonstrated that population of selected oblasts improved their dietary practices. In the intervention areas the share of respondents who reported eating vegetables less than once a day reduced from 66% in 2018 to 52% in 2021 and the share of those who consumed fruits less than once a day dropped from 77% in 2018 to 58% in 2021. It should be noted that the consumption of fruits and vegetables was enhanced in control area as well where the PEN protocol is not implemented. The relation between these variables was statistically significant, X^2^ (1, N = 3,200) = 66.7, *p* < 0.0001. People in intervention area started to consume more vegetables within the 4-year period from 2018 to 2021. A chi-square test of independence was performed to examine the relation between the year of study (baseline or endline) and consumption of fruits among people in intervention area. The relation between these variables was statistically significant, X^2^ (1, N = 3,200) = 200.4, *p* < 0.0001. People in intervention area started to consume more fruits within the 4-year period from 2018 to 2021. The consumption of vegetables and fruits improved both in control and intervention areas, the logistic regressions results, however, illustrate that difference in vegetables and fruit consumption between the baseline and endline studies was significantly stronger in intervention than in control area (see Supplementary Annex Box S1, S2). Stronger improvement in vegetables consumption at endline in intervention area than in control area is indicated by OR = 0.619 which means less chance by 38% (95% CI [0.303, 0.935) a person from intervention area to consume vegetables less than once a day compared to person from control area. Improvement in fruits consumption is indicated by OR = 0.600, which means the odds of consuming fruits less than once a day decreased by 40% (95% CI [0.275, 0.926]) for persons from intervention area at the endline compared to persons from control area.

#### Dietary Salt Intake

The study’s results suggest that people in intervention areas started to consume less salt. Thus, in intervention area, there was an observed reduction in the share of respondents who consumed very salty, salty or medium salty food (from 79% in 2018 to 68% in 2021); always had salt cellar on the table when they had meals (from 38% in 2018 to 26% in 2021) and who drank salty tea (from 7% in 2018 to 4% in 2021). In comparison to the intervention areas, the level of salt consumption was lower in control area in 2018 and reduced for 1% in 2021. The relation between these variables was statistically significant, X^2^ (1, N = 3,200) = 41.6, *p* < 0.0001. People in intervention areas reduced salt intake within the 4-year period from 2018 to 2021. The consumption of salt has reduced both in control and intervention areas, the logistic regression results, however, illustrate that difference in salt intake between the baseline and endline studies was significantly stronger in intervention than in control area (See Supplementary Annex Box S4: OR = 0.624, 95% CI [0.281, 0.967]).

#### Consumption of Animal Fats

The share of respondents who indicated that they have meals with animal fat has slightly reduced both in intervention (from 3.8% to 3.3%) and control area (from 2% to 1.5%) despite the fact that regular viral messages circulated in social media, stating that consumption of animal fat stimulates immunity and resistance to the coronavirus. The relation between these variables was not statistically significant, X^2^ (1, N = 3,200) = 0.582, *p* = 0.445. In this particular element of the KAP study, the reduction in animal fat consumption in intervention area in 2021 compared to 2018 might be obtained by chance.

## Discussion

The results of the KAP study suggest the effectiveness of the PEN protocol in the intervention areas. From a knowledge point of view, the most significant improvement was on knowledge about at least one of NCD-related risk factors, which increased from 61% in 2018 to 87% in 2021 in the intervention areas. The knowledge about the main behavioural risks has increased over the 4 years of the PEN implementation: about the harmful effect of smoking, from 28% to 64%; about eating fat food, from 32% to 68%; about physical inactivity, from 11% to 40%; about harm of alcohol, from 29% to 64%. The same indicators in the control area also improved, but considerably less than in the intervention area: knowledge about the harmful effects of smoking increased from 13% to 34%; about harmful use of alcohol from 15% to 31%; about eating fat food increased from 25% to 30%; about physical inactivity increased from 9% to 22%. Very notable behavioural changes of the population were observed in the intervention areas, where physical activity increased from 23% in 2018 to 35% in 2021; the share of smokers reduced from 22% in 2018 to 20% in 2021; alcohol consumption reduced from 23% in 2018 to 16% in 2021; daily walking at least 30 min improved from 40% to 71%; nutrition practices also improved (vegetable and fruit consumption increased, salt and animal fat intake decreased). These behavioural indicators in the control area also changed: smoking practice has increased from 12% to 15%; alcohol consumption increased from 10% to 11%; daily walking 30 min reduced from 78% to 61%; nutrition practices slightly improved. The difference between baseline and endline data in the intervention areas was statistically significant in all four indicators. All these results confirmed effectiveness of the health education and counselling healthy behaviours within the PEN protocol implementation. Similar KAP studies conducted in Mongolia, China, and Pakistan [9–14] confirmed that the earlier health education began, the more knowledge people had about risk behaviors and the better their health outcomes.

The PEN implementation in Kyrgyzstan was not only within the health system, but also introduced in the secondary schools and universities. Taking into account that a quarter of the respondents were in the age of 18–28, it is assumed that health education introduced in the schools and universities of the intervention area had considerably contributed to the improved indicators. Effectiveness of introduction of the health education at schools were confirmed in several countries [14–18]. Moreover, the health education could be part of a workplace policies as it was suggested in the study conducted in the University in Nigeria [19].

The KAP study period coincided with the same period of COVID-19 outbreak and could have impacted on the study results either in positive or negative way. In 2020, MoH conducted a massive media campaign through radio, TV and social networks to prevent COVID. In parallel to this, social networks spread much of fake and controversial information that smoking and alcohol would help with coping strategies against COVID or that animal fat would increase immunity. The indicator on smoking was a challenge to track because of belief in social media that smokers were protected against COVID. Thus, it was quite difficult both to convince current smokers to give it up as well as to inspire former smokers not to restart smoking. The same goes for alcohol, when it was challenging to convince people about harmful effect of alcohol consumption due to information spread through social media that alcohol could kill the virus. The false belief in protective effect of smoking has led to an increase in tobacco consumption in a number of countries [20]. It should also be noted here that in the control area, the proportion of the daily walking people reduced from 78% in 2018 down to 61% in 2021. This result requires further investigation to detect the reduction reasons and if COVID could have been one of the factors.

It is notable that the consumption of fruits and vegetables had enhanced both in the control area as well as in the intervention area. Such an improvement could be attributed to the pandemic situation for the following reasons 1) people were more concerned about their health condition and tried to pay attention to their diet to strengthen their immune system; 2) because of the lockdown and bans on social gathering, people could save considerably large amounts of money traditionally spent before on wedding, funerals and birthdays and due to lockdown could direct the saved money in a diverse and healthier diet.

The limitations of this study include that within the 4 years of the intervention the COVID-19 pandemic affected the PEN implementation by limiting communication and face to face educational sessions. In addition to this, during the pandemic a very contradictive information was distributed in the social media, such as smokers are protected from COVID, that alcohol kills virus and animal fat improves immunity. All these factors slowed down the PEN implementation and could have a certain influence on the population’s behaviour. However, this is impossible to measure in the scope of this study. In relation to the data collection, all data were self-reported and the distribution of the selected respondents by urban and rural population in intervention and control area was more even during the endline study (21% of the sample in intervention and 22% of the sample in control area lived in urban settlements). As the collected data from interviews are self-reported it is impossible to control the truth of the answers on behavioral changes. Additional limitations were the ethnic, climatic-geographical and cultural differences of the intervention and control areas, which could influence some attitude and behaviour of the population. Namely, it could be that population in the control area was less prone to consume alcohol for religious reasons.

### Conclusion

This study suggests an effectiveness of the PEN protocol in promoting healthy behaviour, improving knowledge and healthy behaviour of the population in rural and urban settings. The implementation of the PEN-2 requires trained nurses at PHC facilities in contexts like Kyrgyzstan where there is a lack of family doctors. To ensure effective implementation of PEN protocol by nurses, the PHC facilities should provide a separate well equipped office for nurses to receive patients, train them and supervise their work. In addition to this, nurses should learn to create and conduct Patient Education Schools as group sessions by type of diseases. During their outreach work, nurses should measure risk factors of patient’s relatives to detect NCDs at early stages. Interventions of the PEN protocol do not require many financial resources since this approach integrated in the daily work of the PHC system and therefore it is affordable for all LMICs. The initial steps to integrate the PEN protocol in the PHC practice require financing for trainings, printouts and supportive monitoring, which in average cost around USD 20 per medical personnel. Thus, it is clearly effective tool to prevent NCDs with long-term impact and at low-cost.

WHO could further advocate for adoption of PEN in other member countries and engage in policy dialogue on promotion based on the PEN protocol implementation experience in the Kyrgyz Republic. Our study provides a practical example that may be used when promoting the adaptation of the PEN-tool in other countries.
