# Supplementary material for: Addressing Non-Communicable Diseases in Primary Healthcare in Kyrgyzstan: A Study on Population’ Knowledge and Behavioral Changes
Source: Int J Public Health. 2023 Jul 4;68:1605381. doi: 10.3389/ijph.2023.1605381 (PMC10353050; doi:10.3389/ijph.2023.1605381)
Supplement: Supplementary file 1 [file DataSheet1.docx]

**Supplementary material**

**of the manuscript**

**Addressing non-communicable diseases in primary health care in Kyrgyzstan: a study on population’ knowledge and behavioral changes**

**Annex 1: Tables**

**Table 1. Characteristics of respondents of the survey sample (Addressing non-communicable diseases in primary health care in Kyrgyzstan: a study on population’ knowledge and behavioral changes; Naryn, Talas, Chui, Issyk-Kul and Djalalabad oblasts, Kyrgyzstan, 2018 and 2021)**

| **Characteristics of respondents** | | **Baseline study in 2018 (n=2000)** | | | | **Endline study in 2021 (n=2000)** | | | |
| --- | --- | --- | --- | --- | --- | --- | --- | --- | --- |
|  |  | Intervention area (n=1600) | | Control area (n=400) | | Intervention area (n=1600) | | Control area (n=400) | |
|  |  | n | % | n | % | n | % | n | % |
| **Gender** | Male | 785 | 49% | 199 | 50% | 791 | 49% | 200 | 50% |
|  | Females | 815 | 51% | 201 | 50% | 809 | 51% | 200 | 50% |
| **Age** | 18-28 | 416 | 26% | 112 | 28% | 352 | 22% | 112 | 28% |
|  | 29-38 | 400 | 25% | 124 | 31% | 464 | 29% | 124 | 31% |
|  | 39-48 | 288 | 18% | 64 | 16% | 288 | 18% | 64 | 16% |
|  | 49-58 | 240 | 15% | 60 | 15% | 224 | 14% | 56 | 14% |
|  | 59 and older | 256 | 16% | 44 | 11% | 272 | 17% | 48 | 12% |
| **Merital status** | Never married | 224 | 14% | 40 | 10% | 192 | 12% | 36 | 9% |
|  | Married | 1232 | 77% | 332 | 83% | 1280 | 80% | 340 | 85% |
|  | Widowed and not remerried | 0 |  | 0 |  | 80 | 5% | 12 | 3% |
|  | Divorced | 144 | 9% | 28 | 7% | 48 | 3% | 12 | 3% |
| **Residing area** | Urban | 304 | 19% | 92 | 23% | 336 | 21% | 88 | 22% |
|  | Rural | 1296 | 81% | 312 | 78% | 1264 | 79% | 312 | 78% |
| **Household size** | Mean | 4.8 |  | 5.8 |  | 4.9 |  | 5.9 |  |
|  | Standard deviation | 1.97 |  | 2.30 |  | 1.95 |  | 2.29 |  |
|  | Minumum | 1 |  | 1 |  | 1 |  | 1 |  |
|  | Maximum | 12 |  | 20 |  | 12 |  | 18 |  |
| **Education** | Basic general or lower | 112 | 7% | 32 | 8% | 80 | 5% | 28 | 7% |
|  | Secondary general | 592 | 37% | 192 | 48% | 608 | 38% | 184 | 46% |
|  | Primary professional | 80 | 5% | 12 | 3% | 112 | 7% | 28 | 7% |
|  | Secondary professional | 368 | 23% | 88 | 22% | 384 | 24% | 80 | 20% |
|  | Non-finished higher | 64 | 4% | 8 | 2% | 80 | 5% | 24 | 6% |
|  | Higher or PhD | 352 | 22% | 72 | 18% | 352 | 22% | 60 | 15% |
| **Employ-ment** | I am employed on a regular, long-term basis | 272 | 17% | 56 | 14% | 336 | 21% | 72 | 18% |
|  | Periodically work on short-term, one-time employment contracts, working temporarily employed in seasonal work | 112 | 7% | 24 | 6% | 112 | 7% | 16 | 4% |
|  | I am private entrepreneur, businessman | 80 | 5% | 44 | 11% | 64 | 4% | 36 | 9% |
|  | I am engaged in farming and / or livestock | 192 | 12% | 20 | 5% | 224 | 14% | 40 | 10% |
|  | Self-employed | 64 | 4% | 20 | 5% | 32 | 2% | 24 | 6% |
|  | Non-working pensioners | 240 | 15% | 44 | 11% | 224 | 14% | 44 | 11% |
|  | Non-working student | 48 | 3% | 8 | 2% | 48 | 3% | 12 | 3% |
|  | Housewife, on maternity leave | 368 | 23% | 104 | 26% | 304 | 19% | 52 | 13% |
|  | No, I don't work because of disability/ health reasons | 32 | 2% | 8 | 2% | 16 | 1% | 4 | 1% |
|  | No, I am unemployed | 192 | 12% | 76 | 19% | 240 | 15% | 100 | 25% |

**Table 2. Knowledge about NCD risk factors (% of respondents mentioning the risk factor) (Addressing non-communicable diseases in primary health care in Kyrgyzstan: a study on population’ knowledge and behavioral changes; Naryn, Talas, Chui, Issyk-Kul and Djalalabad oblasts, Kyrgyzstan, 2018 and 2021)***

| **NCD risk factors** | **Baseline survey in 2018 (n=2000)** | | | | **Endline survey in 2021 (n=2000)** | | | |
| --- | --- | --- | --- | --- | --- | --- | --- | --- |
|  | Intervention area (n=1600) | | Control area (n=400) | | Intervention area (n=1600) | | Control area (n=400) | |
|  | n | % | n | % | n | % | n | % |
| **Stress** | 832 | 52% | 180 | 45% | 912 | 57% | 296 | 74% |
| **Eating fat food** | 512 | 32% | 100 | 25% | 1088 | 68% | 120 | 30% |
| **Use of alcohol** | 464 | 29% | 60 | 15% | 1024 | 64% | 124 | 31% |
| **Smoking** | 448 | 28% | 52 | 13% | 1024 | 64% | 136 | 34% |
| **Overweight** | 304 | 19% | 64 | 16% | 528 | 33% | 168 | 42% |
| **Eating high sugar food** | 288 | 18% | 52 | 13% | 944 | 59% | 52 | 13% |
| **Eating salty food/drinks** | 288 | 18% | 92 | 23% | 976 | 61% | 88 | 22% |
| **Physical inactivity** | 176 | 11% | 36 | 9% | 640 | 40% | 88 | 22% |
| **Genetic factors** | 128 | 8% | 28 | 7% | 112 | 7% | 64 | 16% |
| **High blood pressure** | 128 | 8% | 88 | 22% | 128 | 8% | 92 | 23% |
| **Ecological problems, pollution** | 112 | 7% | 8 | 2% | 32 | 2% | 12 | 3% |
| **Age risk ( more 40 years old)** | 80 | 5% | 8 | 2% | 32 | 2% | 36 | 9% |
| **Bad quality of products** | 80 | 5% | 4 | 1% | 32 | 2% | 32 | 8% |
| **Unhealthy diet** | 64 | 4% | 0 |  | 32 | 2% | 8 | 2% |
| **Poverty** | 32 | 2% | 4 | 1% | 0 | 0% | 0 | 0% |
| **Other** | 112 | 7% | 16 | 4% | 48 | 3% | 20 | 5% |
| **Does not apply/I do not know** | 240 | 15% | 140 | 35% | 112 | 7% | 16 | 4% |
| **% of respondents who know at least 1 NCD-related risk factors: overweight, eating high sugar food, eating salty food/drinks, eating fat food, smoking, use of alcohol, and physical inactivity** | 970 | 61% | 191 | 48% | 1390 | 87% | 273 | 68% |

**Table 3. Change of physical activity among population (Addressing non-communicable diseases in primary health care in Kyrgyzstan: a study on population’ knowledge and behavioral changes;Naryn, Talas, Chui, Issyk-Kul and Djalalabad oblasts, Kyrgyzstan, 2018 and 2021)***

| **Physical inactivity (not walking 30 minutes per day) by type of settlement and gender** | | **Baseline study in 2018 (n=2000)** | | | | **Endline study in 2021 (n=2000)** | | | |
| --- | --- | --- | --- | --- | --- | --- | --- | --- | --- |
|  |  | Intervention area (n=1600) | | Control area (n=400) | | Intervention area (n=1600) | | Control area (n=400) | |
|  |  | n | % | n | % | n | % | n | % |
| Type of settlement | Urban | 206 | 67% | 6 | 7% | 118 | 36% | 41 | 46% |
|  | Rural | 751 | 58% | 83 | 27% | 339 | 27% | 114 | 37% |
| Gender | Male | 437 | 56% | 39 | 20% | 204 | 26% | 75 | 70% |
|  | Females | 520 | 64% | 50 | 25% | 253 | 38% | 80 | 40% |
| **Total** | | 957 | 60% | 89 | 22% | 457 | 29% | 155 | 39% |
| **Go in for sport** | Yes | 368 | 23% | 128 | 32% | 512 | 32% | 140 | 35% |
|  | No | 1232 | 77% | 272 | 68% | 1088 | 68% | 260 | 65% |
| **Number of days per week walk no less than 30 min** | Daily | 640 | 40% | 312 | 78% | 1136 | 71% | 244 | 61% |
|  | 5-6 days per week | 128 | 8% | 28 | 7% | 96 | 6% | 20 | 5% |
|  | 3-4 days per week | 256 | 16% | 32 | 8% | 192 | 12% | 52 | 13% |
|  | 1-3 days per week | 272 | 17% | 20 | 5% | 176 | 11% | 84 | 21% |
|  | None | 304 | 19% | 12 | 3% | 0 | 0% | 0 | 0% |

**Table 4. Change of practice among population (smoking, alcohol, dietary practice) by type of settlement and gender (Addressing non-communicable diseases in primary health care in Kyrgyzstan: a study on population’ knowledge and behavioral changes; Naryn, Talas, Chui, Issyk-Kul and Djalalabad oblasts, Kyrgyzstan, 2018 and 2021)**

| **Prevalence of smoking** | | | **Baseline study in 2018 (n=2000)** | | | | | | | | | | **Endline study in 2021 (n=2000)** | | | | | | | | | | | |
| --- | --- | --- | --- | --- | --- | --- | --- | --- | --- | --- | --- | --- | --- | --- | --- | --- | --- | --- | --- | --- | --- | --- | --- | --- |
|  |  |  | Intervention area (n=1600) | | | | Control area (n=400) | | | | | | Intervention area (n=1600) | | | | | | Control area (n=400) | | | | | |
|  |  |  | n | % | | | n | | | % | | | n | | | % | | | n | | | | % | |
| **Settlement type** | Urban | | 59 | 19% | | | 9 | | | 10% | | | 67 | | | 20% | | | 16 | | | | 18% | |
|  | Rural | | 290 | 22% | | | 40 | | | 13% | | | 258 | | | 20% | | | 42 | | | | 14% | |
| **Gender** | Male | | 335 | 43% | | | 48 | | | 24% | | | 304 | | | 38% | | | 57 | | | | 29% | |
|  | Females | | 14 | 2% | | | 1 | | | 0% | | | 21 | | | 3% | | | 1 | | | | 1% | |
| **Total** | | | 349 | 22% | | | 49 | | | 12% | | | 325 | | | 20% | | | 58 | | | | 15% | |
| **Prevalence of alcohol consumers**  (% of respondents who consumed alcohol within the past 30 days) | | | **Baseline study in 2018 (n=2000)** | | | | | | | | | | | | **Endline study in 2021 (n=2000)** | | | | | | | | | |
|  |  |  | Intervention area (n=1600) | | | | | | Control area (n=400) | | | | | | Intervention area (n=1600) | | | | | | Control area (n=400) | | | |
|  |  |  | n | | | % | | | n | | | % | | | n | | | % | | | n | | | % |
| **Settlement type** | | Urban | 71 | | | 23% | | | 4 | | | 4% | | | 62 | | | 19% | | | 14 | | | 16% |
|  |  | Rural | 290 | | | 22% | | | 35 | | | 11% | | | 199 | | | 16% | | | 29 | | | 9% |
| **Gender** | | Male | 272 | | | 35% | | | 32 | | | 16% | | | 185 | | | 23% | | | 36 | | | 18% |
|  |  | Females | 89 | | | 11% | | | 7 | | | 3% | | | 75 | | | 9% | | | 7 | | | 4% |
| **Total** | | | 361 | | | 23% | | | 39 | | | 10% | | | 260 | | | 16% | | | 43 | | | 11% |
| **Dietary practice:**  **Fruits and vegetables consumption** | | | **Baseline study in 2018 (n=2000)** | | | | | | | | | | | **Endline study in 2021 (n=2000)** | | | | | | | | | | |
|  |  |  | Intervention area (n=1600) | | | | | Control area (n=400) | | | | | | Intervention area (n=1600) | | | | | | Control area (n=400) | | | | |
|  |  |  | n | | % | | | n | | | % | | | n | | | % | | | n | | % | | |
| Consume vegetables less than once a day | | | 1056 | | 66% | | | 244 | | | 61% | | | 832 | | | 52% | | | 212 | | 53% | | |
| Consume fruits less than once a day | | | 1232 | | 77% | | | 268 | | | 67% | | | 928 | | | 58% | | | 216 | | 54% | | |
| **Salt intake** | | |  | |  | | |  | | |  | | |  | | | | | | | | | | |
| ery salty, salty or medium salty food consumption | | | 1264 | | 79% | | | 284 | | | 71% | | | 1088 | | | 68% | | | 280 | | 70% | | |
| Always have salt cellar on the table when they have meal | | | 608 | | 38% | | | 68 | | | 17% | | | 416 | | | 26% | | | 36 | | 9% | | |
| Drinking salty tea | | | 112 | | 7% | | | 0 | | | 0 | | | 64 | | | 4% | | | 0 | | 0 | | |
| **Consumption of animal fat** | | | 61 | | 3,8% | | | 8 | | | 2% | | | 53 | | | 3,3% | | | 6 | | 1,5% | | |

**Annex 2: Questionnaire**

**KAP survey on population knowledge on Noncommunicable diseases**

**and its risk factors**

(Addressing non-communicable diseases in primary health care in Kyrgyzstan: a study on population’ knowledge and behavioral changes; Naryn, Talas, Chui, Issyk-Kul and Djalalabad oblasts, Kyrgyzstan, 2018 and 2021)

| Questionnaire number |  |  |  |  |
| --- | --- | --- | --- | --- |

| Date of interview | Date | | Month | | Year | | | |
| --- | --- | --- | --- | --- | --- | --- | --- | --- |
|  |  |  |  |  |  |  |  |  |

Hello! We are conducting a survey on health issues in the Chui, Talas, Naryn, Issyk-Kul and Jalal-Abad regions of the Kyrgyz Republic. Your family was randomly selected. In total, we will have more than 1,000 such surveys in 5 regions of Kyrgyzstan. The answers to these questions cannot be right or wrong, good or bad. More important is the fact that your answers were truthful, sincere and reflected your experience and what you really think. It will take about 15 minutes to complete the survey questionnaire. Participation in this survey is voluntary. You have the right to terminate the survey at any time and not answer certain questions in the questionnaire. We guarantee you that your answers will be used only for research purposes. Your name and address will not be displayed in any documents. Your participation is very important for our research. We really appreciate your experience and participation.

1) Do you have any questions?

2) Do you agree to participate in the survey?

INTERVIEWER: Continue the interview if the respondents agree to participate.

**In the beginning few questions about you:**

| **В1. Gender**  *INTERVIEWER: you can cancel the answer, without asking the question.* | |
| --- | --- |
| 1 | Mail |
| 2 | Female |

| **В2. Date of birth?** |  | day |  | month |  | year |
| --- | --- | --- | --- | --- | --- | --- |

| **В3. Your family status?**  *INTERVIEWER: Only one answer is possible.* | |
| --- | --- |
| 1 | Never married |
| 2 | Married |
| 3 | Widow, widower, not remarried |
| 4 | Divorced |
| *99* | *No answer* |

| **В4. What is your education?**  *INTERVIEWER: Please highlight highest education level. One answer is possible.* | |
| --- | --- |
| 1 | Do not have education |
| 2 | Preschool education |
| 3 | Primary school (finished first 3 or 4 years of school education) |
| 4 | Basic secondary education (finished 9 years) |
| 5 | General secondary education (finished 11 years) |
| 6 | Vocational professional training (lyceum) |
| 7 | Secondary special education (college) |
| 8 | Not finished high education |
| 9 | High education (diploma of specialist, minimum 5 years of education, bachelor or magister) or candidate of science (PhD) |
| *99* | *No answer* |

| **В5. Are you working currently? We are interested in paid and unpaid job in family business, farm, training at specific master classes or other skills development processes**  *INTERVIEWER: One answer is possible.* | |
| --- | --- |
| 1 | I work (oral or written contract), I have a regular, long-term job (with the exception of maternity leave or parental leave) |
| 2 | Periodically I work under short-term one-time employment contracts, I work temporarily at seasonal jobs |
| 3 | I am a private entrepreneur, businessman |
| 4 | I am engaged in agriculture and/or animal husbandry (this means only those who live off their/leased land and/or livestock) |
| 5 | Self-employed (small business, such as handicrafts, "shuttles", etc.) |
| 6 | Non-working pensioners (including early retirement), do not engage in any activity |
| 7 | Unemployed student |
| 8 | Housewife, on maternity leave |
| 9 | No, I don't work because of disability/health reasons |
| 10 | No, I'm unemployed |
| *99* | No answer |

| **В6. How would you describe your family's financial situation? Under "family" I mean all the people who live together with you and have common incomes and expenses.**  INTERVIEWER: One answer is possible. | |
| --- | --- |
| 1 | We don't even have enough for food. |
| 2 | We have enough money for food, but it is difficult to buy clothes. |
| 3 | We can afford food and clothes, and we can save a little money, but we can't afford expensive things like a refrigerator or an automatic washing machine. |
| 4 | We can afford some expensive things like a refrigerator or an automatic washing machine, but we can't afford to buy a car. |
| 5 | We can afford everything except real estate, for example, cottages or apartments. |
| 6 | We have no financial difficulties. If necessary, we can buy a cottage or an apartment. |
| *99* | No answer |

| **В7. How many people are leaving together in your family, including you?** |  | people |
| --- | --- | --- |

| **В8. What is your household's monthly income from all sources? Include all monetary incomes of all family members, i.e. salaries, pensions, scholarships and other monetary receipts, in soms.** |  | soms |
| --- | --- | --- |

| **В9. How you would assess your health in general?**  *INTERVIEWER: One answer is possible.* | |
| --- | --- |
| 1 | Very good |
| 2 | Good |
| 3 | Satisfactory |
| 4 | Bad |
| 5 | Very bad |
| 99 | *I don’t know or not applicable* |

| **В10. Do you have any chronic diseases?**  *INTERVIEWER: One answer is possible.* | | |
| --- | --- | --- |
| 1 | Yes | *Move to the question В10/1* |
| 2 | No | *Move to the question В11* |

| **В10/1. If yes, please, indicate which once**  *INTERVIEWER: One answer is possible for one row.* | | |
| --- | --- | --- |
|  | **Yes** | **No** |
| 1. Cancer | 1 | 2 |
| 1. Hypertension | 1 | 2 |
| 1. Diabetes | 1 | 2 |
| 1. Cardiovascular diseases | 1 | 2 |
| 1. Other, please indicate which one | 1 | 2 |

**Now we move to the questions on some diseases**

| **В11. How do you think, what kind of factors result in diabetes, hypertension, cancer, cardiovascular diseases?**  *INTERVIEWER: Do not read all listed answers. Several answers possible.* | |
| --- | --- |
| 1 | Overweight |
| 2 | Consumption food with high level of sugar |
| 3 | Consumption salty products / drinks |
| 4 | Consumption fat food |
| 5 | Stress |
| 6 | Smoking |
| 7 | Alcohol consumption |
| 8 | High blood pressure |
| 9 | Age risk (over 40 years old) |
| 10 | Genetic (hereditary) factors |
| 11 | Low physical activity |
| 88 | Other (indicate which) |
| 99 | *Not applicable / Do not know* |

| **В12. Do you think that diabetes, hypertension, cancer, cardiovascular diseases can be prevented?**  *INTERVIEWER: One answer is possible.* | | |
| --- | --- | --- |
| 1 | Yes | - *Move to the question В12/1* |
| 2 | No | - *Move to the question В13* |
| 99 | *Not applicable / Do not know* | - *Move to the question В13* |

| **В12/1. If yes, please, tell me, how?**  *INTERVIEWER: Do not read the list of answers. Several answers possible. If responded will answer “diet”, ask to clarify what she/he means.* | |
| --- | --- |
| 1 | Do not smoke |
| 2 | Do not drink alcohol excessively |
| 3 | Eat/drink food/drinks with low salt content |
| 4 | Eating food low in animal fats |
| 5 | Eating low-sugar foods |
| 6 | Eat fruits and vegetables regularly |
| 7 | Exercise regularly |
| 8 | Monitor blood pressure |
| 9 | Control your own weight |
| 10 | Check cholesterol levels |
| 11 | Regularly undergo a medical examination |
| 12 | Check your blood sugar level |
| 13 | Manage stress (less stress) |
| 88 | Other (specify) |
| 99 | Not applicable/I don't know |

| **В13. From what sources do you know about diabetes, hypertension, cancer, cardiovascular diseases and their prevention?**  *INTERVIEWER: Several answers possible.* | |
| --- | --- |
| 1 | Family member |
| 2 | Educational institution (school, college, university) |
| 3 | Colleagues |
| 4 | Friends |
| 5 | Doctors |
| 6 | Nurses\midwifes |
| 7 | Television |
| 8 | Radio |
| 9 | Internet |
| 10 | Information campaign/events |
| 11 | Informational materials (leaflets, booklets, billboards) |
| 12 | Newspapers/magazines |
| 13 | Village Health Committees |
| 88 | Other (specify) |
| 99 | Not applicable/I don't know |

| **В14. Do you think you need more information about diabetes, hypertension, cancer, cardiovascular diseases?**  *INTERVIEWER: Only one answer is possible.* | | |
| --- | --- | --- |
| 1 | Yes | - *Move to the next question В14/1* |
| 2 | No | - *Move to the next question В15* |

| **В14/1. From which source/s would you like to learn more about diabetes, hypertension, cancer, cardiovascular diseases and their prevention?**  *INTERVIEWER: Several answers possible.* | |
| --- | --- |
| 1 | Family member |
| 2 | Educational institution (school, college, university) |
| 3 | Colleagues |
| 4 | Friends |
| 5 | Doctors |
| 6 | Nurses/midwifes |
| 7 | Television |
| 8 | Radio |
| 9 | Internet |
| 10 | Information campaign/events |
| 11 | Informational materials (leaflets, booklets, billboards) |
| 12 | Newspapers/magazines |
| 13 | Village Health Committees |
| 88 | Other (specify) |
| 99 | Not applicable/I don't know |

| **В15. In your opinion, who is responsible for the prevention of diabetes, hypertension, cancer, and cardiovascular diseases?**  *INTERVIEWER: Do not read the list of answers. Several answers possible.* | |
| --- | --- |
| 1 | People themselves |
| 2 | Healthcare workers in medical institutions |
| 3 | Educational institutions |
| 4 | Government |
| 5 | Employers |
| 6 | Community, civil society |
| 88 | Other (specify) |
| 99 | Not applicable/I don't know |

**Now, few questions about your behavior.**

| **B16. Do you sport?**  *INTERVIEWER: One answer is possible.* | | |
| --- | --- | --- |
| 1 | Yes | - *Move to the question B17* |
| 2 | No | - *Move to the question B16/1* |

| **B16/1. Are you ready, or wish to sport?**  *INTERVIEWER: Only one answer is possible. .* | | |
| --- | --- | --- |
| 1 | Yes | - *Move to the question. B17* |
| 2 | No | - *Move to the question. B16/2* |
| 99 | *Not applicable/I do not know* | - *Move to the question. B16/2* |

| **В16/2. If No, then why?**  *INTERVIEWER: Several answers possible.* | |
| --- | --- |
| 1 | It is expensive |
| 2 | It's a long way from my house |
| 3 | I don't have time |
| 4 | I don't think I need it. |
| 5 | I do exercises at home |
| 88 | Other (specify) |
| 99 | Not applicable/I don't know |

| **B17. Have you ever been given advice/advice by health care workers to do physical exercises?**  *INTERVIEWER: Only one answer is possible. .* | | |
| --- | --- | --- |
| 1 | Yes | - *Move to the question. B17/1* |
| 2 | No | - *Move to the question. B18* |
| 99 | *Not applicable / I do not know* | - *Move to the question. B18* |

| **B17/1. If Yes, then who?**  *INTERVIEWER: Several answers possible.* | |
| --- | --- |
| 1 | Your family doctor |
| 2 | Your nurse\paramedic |
| 3 | Doctors of the district/regional hospital |
| 4 | Doctors at republican hospitals in Bishkek |
| 5 | BODY SHOP workers |
| 6 | Private doctors |
| 88 | Other (specify) |
| 99 | Not applicable/I don't know |

| **B18. How many minutes a day do you walk?** | ___ Minutes |
| --- | --- |

| **B19. Do you currently smoke? Have you ever smoked?**  *INTERVIEWER: Only one answer is possible. .* | | |
| --- | --- | --- |
| 1 | Yes, I smoke | - *Move to the question. B20* |
| 2 | I used to smoke, but stopped less than a year ago | - *Move to the question. B19/1* |
| 2 | I used to smoke, but stopped a year or more ago | - *Move to the question. B819/1* |
| 3 | I don't smoke | - *Move to the question. B20* |

| **B19/1. If you quit/stop smoking, Who advised you?**  *INTERVIEWER: Several answers possible.* | |
| --- | --- |
| 1 | Your family doctor |
| 2 | Your nurse\paramedic |
| 3 | Family |
| 4 | Friends |
| 5 | I have seen/read some informational materials about the dangers of smoking |
| 6 | Health Promotion Units / Village Health Committees |
| 88 | Other (specify) |
| 99 | Not applicable/I don't know |

| **B20. Have you consumed alcohol (such as vodka, beer, wine, cognac) within the past 30 days?**  *INTERVIEWER: Only one answer is possible. .* | | |
| --- | --- | --- |
| 1 | Yes | - *Move to the question. B20/1* |
| 2 | No | - *Move to the question. B21* |

| **B20/1. Over the past 30 days, how often have you had at least one alcoholic drink?**  *INTERVIEWER: Only one answer is possible.* | |
| --- | --- |
| 1 | Every day |
| 2 | 5-6 day a week |
| 3 | 1-4 day a week |
| 4 | 1-3 day a month |
| 5 | Less than once in a month |

| **B21. Do you know your weight?**  *INTERVIEWER: Only one answer is possible.* | |
| --- | --- |
| 1 | Yes |
| 2 | No |

| **B22. How often you check your weight?**  *INTERVIEWER: Only one answer is possible.* | |
| --- | --- |
| 1 | Never |
| 2 | Once a year |
| 3 | Once a month |
| 4 | Twice in a month |
| 5 | Every week |
| 6 | Every day |

| **B23. If you have overweight, did you try to loose weight?**  *INTERVIEWER: Only one answer is possible.* | |
| --- | --- |
| 1 | I have normal weight |
| 2 | Yes, I tried to loose |
| 3 | No, I never tried to loose |

| **B24. During the last 30 days, how many times a day did you usually eat vegetables (cabbage, cucumber, carrots, tomatoes, lettuce)?**  *INTERVIEWER: Only one answer is possible. .* | |
| --- | --- |
| 1 | I haven't eaten vegetables in the last 30 days |
| 2 | Less than 7 times a week |
| 3 | 1 time per day |
| 4 | 2 times a day |
| 5 | 3 times a day or more |
| **B25. DURING THE LAST 30 DAYS, HOW MANY TIMES A DAY DID YOU USUALLY EAT FRUITS (APPLES, ORANGES, BANANAS, GRAPES)?**  *INTERVIEWER: Only one answer is possible.* | |
| 1 | I haven't eaten fruit in the last 30 days |
| 2 | Less than 7 times a week |
| 3 | 1 time per day |
| 4 | 2 times a day |
| 5 | 3 times a day or more |

| **B26. YOU ALWAYS HAVE A SALT SHAKER ON THE TABLE WHEN YOU EAT?**  *INTERVIEWER: Only one answer is possible.* | |
| --- | --- |
| 1 | Yes |
| 2 | No |

| **B27. How salty do you think the food you usually eat is?**  *INTERVIEWER: Only one answer is possible.* | |
| --- | --- |
| 1 | Very salty |
| 2 | Salty |
| 3 | Average |
| 4 | Slightly salty |
| 5 | Unsalted in general |
| 99 | Not applicable/I don't know |

| **B28. Do you drink tea with salt?**  *INTERVIEWER: Only one answer is possible.* | |
| --- | --- |
| 1 | Yes |
| 2 | No |

| **B29. How many days a week do you use the following fats?** | |
| --- | --- |
|  | *INTERVIEWER: Indicate number of days from 0 to 7* |
| 1. Butter / cooked butter |  |
| 1. Cream |  |
| 1. Vegetable oil |  |
| 1. Animal fat |  |
| 1. Fat meat |  |
| 1. Margarine |  |
| 1. Mayonnaise |  |
| 1. Fat sausage |  |

| **B30. How many days do you eat this type of meat?** | |
| --- | --- |
|  | *INTERVIEWER: Indicate number of days from 0 to 7* |
| 1. Horse meat |  |
| 1. Beef |  |
| 1. Lamb |  |
| 1. Chicken meat |  |
| 1. Goat meat |  |
| 1. Yak meat |  |

**Annex 3:** Summary of binary logistic regressions results used in the statistical analysis (Addressing non-communicable diseases in primary health care in Kyrgyzstan: a study on population’ knowledge and behavioral changes; Naryn, Talas, Chui, Issyk-Kul and Djalalabad oblasts, Kyrgyzstan, 2018 and 2021)
